# Allergic diseases of the skin and drug allergies – 2005. Common causes of allergic contact dermatitis in Kuwait

**DOI:** 10.1186/1939-4551-6-S1-P95

**Published:** 2013-04-23

**Authors:** Mona Al-Ahmad, Nermina Arifohdzic, Naser Fakim

**Affiliations:** 1 Allergy Department, Al-Rashed Allergy Center, Kuwait; 2 Al-Rashed Allergy Center, Kuwait

## Background

Allergic contact dermatitis is caused by a variety of reagents.

## Methods

We looked into the eliciting reagents causing allergic contact dermatitis among patients referred to a skin allergy clinic in Kuwait.

## Results

Total of 153 patients with contact dermatitis were enrolled. Each patient was patch tested using (TRUE Test). The patch was removed after 48 hours and read after 96 hours. A positive reaction was scored according to the standard scoring system recommended by the international group. Mean age was 39.9 (SD 13.84) years old. Females were 78 ( 51%). Majority were Kuwaiti patients 76.5%. Occupation enquiry showed that 57.5% of patients had a desk job, while 23.5% were housewives, and 11.8% were students. Hand contact dermatitis was the most common indicator 45.1% for testing, followed by body and eyelid dermatitis, and hair dye 20.9%, 7.2, and 7.2%% respectively. History of contact to jewels or perfumes was only 2.6% and 3.3% respectively. The test was positive to at least one allergen in 94 (61.4%), with 27.5% having a strong (++) positive reaction, and 33.3% having an extreme (+++) positive reaction. P-phenylendiamine was the leading contact allergen in 31 patients (20.3%), followed by Nickel sulphate in 15 patients (9.8%), Thiomersal in 11 patients (7.2%), Wool alcohols in 6 patients (3.9%), Potassium dichromate in 6 (3.6%), and Epoxy resin in 5 (3.3%).

## Conclusions

The nature of Allergens eliciting contact dermatitis in Kuwait is surprisingly similar to that of the western countries, in which hair dyes reagents and metals are a leading sensitize.

